# Nucleotide-Specific Contrast for DNA Sequencing by Electron Spectroscopy

**DOI:** 10.1371/journal.pone.0154707

**Published:** 2016-05-05

**Authors:** Marian Mankos, Henrik H. J. Persson, Alpha T. N’Diaye, Khashayar Shadman, Andreas K. Schmid, Ronald W. Davis

**Affiliations:** 1 Electron Optica Inc., 1000 Elwell Court #110, Palo Alto, California, 94303, United States of America; 2 Stanford Genome Technology Center, Stanford University School of Medicine, 855 California Avenue, Palo Alto, CA, 94304, United States of America; 3 NCEM, Molecular Foundry, Lawrence Berkeley National Laboratory, 1 Cyclotron Road, Berkeley, CA, 94720, United States of America; SPECS Surface Nano Analysis GmbH, GERMANY

## Abstract

DNA sequencing by imaging in an electron microscope is an approach that holds promise to deliver long reads with low error rates and without the need for amplification. Earlier work using transmission electron microscopes, which use high electron energies on the order of 100 keV, has shown that low contrast and radiation damage necessitates the use of heavy atom labeling of individual nucleotides, which increases the read error rates. Other prior work using scattering electrons with much lower energy has shown to suppress beam damage on DNA. Here we explore possibilities to increase contrast by employing two methods, X-ray photoelectron and Auger electron spectroscopy. Using bulk DNA samples with monomers of each base, both methods are shown to provide contrast mechanisms that can distinguish individual nucleotides without labels. Both spectroscopic techniques can be readily implemented in a low energy electron microscope, which may enable label-free DNA sequencing by direct imaging.

## Introduction

Significant demand exists for the development of high throughput technologies capable of extremely low-cost, high quality DNA sequencing. Established sequencing technologies based on capillary array electrophoresis and cyclic array sequencing offer such analytical capability, and currently marketed 2nd generation sequencers are delivering information at a cost of less than $5,000/genome. One major drawback is that these technologies typically identify only 10–100 bases out of the 3 billion base pairs in the human genome in a given sequence segment or read. Another drawback is the relatively large raw read error rate. Important applications like de novo sequencing assembly, determination of point mutations, differentiation of closely related species, and targeted resequencing require low error rates. Furthermore, the complex repetitive nature of DNA makes it costly, time consuming, and in some cases impossible to accurately reassemble the complete sequence from short reads.

Transmission Electron Microscopy (TEM) has been explored for imaging long DNA segments [1,2] by utilizing high electron energies (80–300 keV) to achieve sub-nanometer resolution. The high impact energy, however, not only produces radiation damage, it necessitates the use of heavy atom labels to provide contrast in the image of the nucleotides. The radiation damage limits the electron dose and thereby the throughput. Furthermore, the complications associated with reliably labeling the bases lead to significant read errors.

Scattering electron beams with significantly lower energies has been shown to prevent radiation damage to biological molecules. For example, experimental work [3–5] carried out by Fink’s group has demonstrated that DNA withstands a radiation dose of 10^8^ electrons/nm^2^ accumulated over more than one hour with impact energies from 60 to 230 eV. The absence of radiation damage associated with low impact energies motivates our goal to establish the feasibility of nucleotide-specific contrast mechanisms that may enable the sequencing of DNA by electron imaging techniques. If sufficient contrast can be achieved then, in principle, sub-nanometer DNA sequence images may be achievable without the need for heavy atom labeling. The elimination of labels not only simplifies the preparation of the DNA strand, but it eliminates the errors associated with attaching the labels and correlating the nucleotides to their labels. Amplification is not needed, which eliminates another source of error in sequencing. Furthermore, an imaging method could enable long read lengths, which reduce the computational complexity and uncertainty associated with stitching the segments to assemble the full sequence.

In a prior study we focused on establishing nucleotide-specific variations of low energy electron reflectivity [6]. In a second study we noted that preliminary results on a single set of samples suggested the possibility that two well-established spectroscopic techniques, X-ray Photoelectron Spectroscopy (XPS) and Auger electron spectroscopy (AES), may be applicable to enable DNA imaging [7].

**Fig 1:**
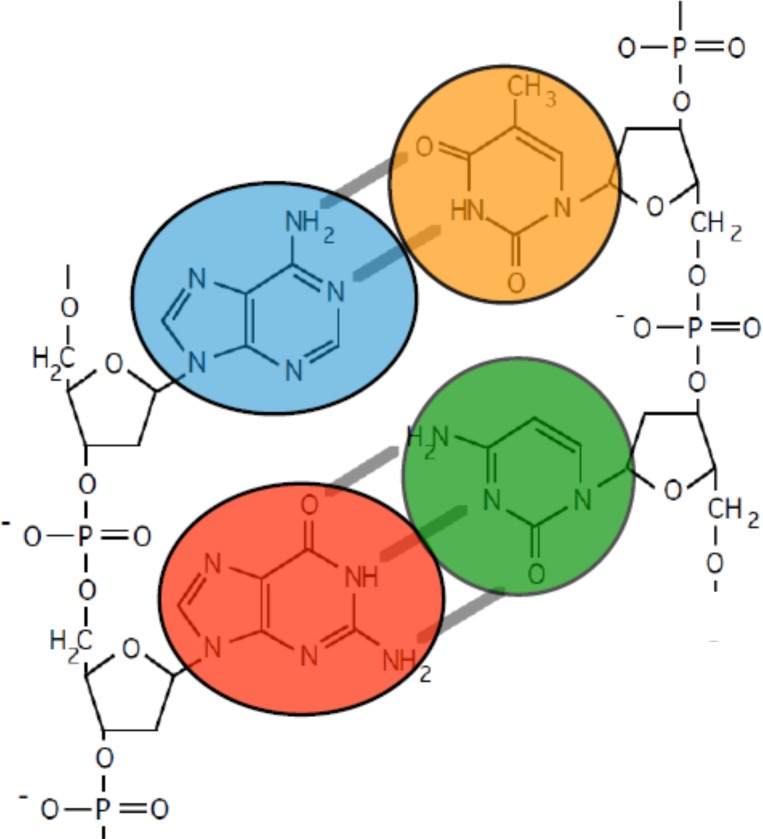
Composition and elemental ratios of DNA bases.

Here we present detailed measurements analyzing the possibility to utilize AES and XPS signals to detect nucleotide-specific contrast. We have utilized specimens with oligomers containing only one of the four single bases (Adenine (A), Cytosine (C), Guanine (G), or Thymine (T)). It is noted that nitrogen is present in the nucleobases and absent in the phosphate-sugar backbone. Furthermore, the purines (A and G) possess more nitrogen than their complements (T and C) because of their imidazole ring. Fig 1 lists the elemental compositions (not including hydrogen) and ratios of key elements for the four nucleotides. The C and T bases can be unambiguously identified by their nitrogen content alone (3 and 2 atoms, respectively). On the other hand, the bases A and G both have 5 nitrogen atoms and cannot be identified by their nitrogen content. However, the state of nitrogen in nitrogen-to-carbon bonds differ for the two bases (single versus double). Furthermore, the A and G bases differ in the number of oxygen atoms. This allows discrimination of the four bases by comparing the O/N ratio of the four bases. The identification of individual bases by comparing C/O ratio is, in principle, also possible although ubiquitous carbon contaminations can obstruct such efforts [8]. Preliminary XPS and AES results on a single set of samples have been reported [7]. Inspired by encouraging signals reported in Ref. 7, we substantiate the presence of base-specific contrast on several sets of samples, varying in oligomer length, purification and film thickness. In addition, we have performed quantitative analysis of the individual spectra that supports our explanation of the base-specific contrast. In the following sections we present experimental data showing that the elemental ratios and bonding states can be used to distinguish the individual DNA bases.

## Results

### X-ray Photoelectron Spectroscopy (XPS)

**Fig 2:**
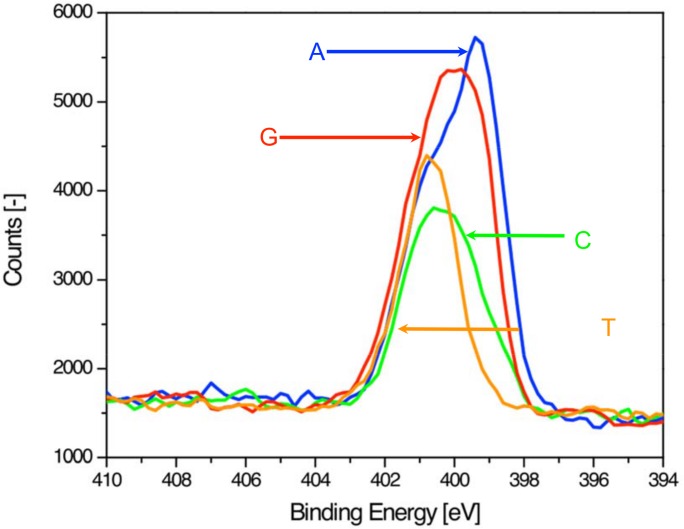
High-resolution nitrogen 1s XPS spectra for single-stranded homopolymeric 20mers.

We used XPS to analyze individual samples of homopolymeric 20mers that were prepared by electrostatic adsorption onto gold coated surfaces modified with aminoundecanethiol. DNA films were tailored to a thickness larger than the escape depth of the photoemitted electrons to minimize signal contributions from both the aminoundecanethiol layer and the gold surface. Survey spectra were obtained to monitor the coverage of DNA and the presence of any contaminants. Subsequently we performed high resolution measurements of the expected DNA elements, i.e. phosphor, nitrogen, oxygen and carbon. Phosphor and nitrogen are particularly interesting candidates for positive identification of DNA because these elements are seldom misrepresented by surface contaminations from sample preparation or handling [8]. Phosphor is present in the phosphate-sugar backbone of DNA at a constant level for all samples (1 phosphate group per base). Nitrogen on the other hand is only present in the nucleobase and varies in content and binding state for each of the 4 nucleotides. Consequently we used the phosphor 2p signal for normalization (S1 Fig) of all samples and the nitrogen 1s spectra for quantitative analysis. Fig 2 shows an overlay of the individual nitrogen 1s spectra, which clearly distinguish the purine bases (A and G) from the pyrimidine bases (C and T): the larger nitrogen content in the purines (5 nitrogen atoms per base) results in larger and wider nitrogen peaks when compared to the pyrimidines (C and T, which have 3 and 2 nitrogen atoms per base, respectively). For detailed quantitative analysis of the samples, the elemental content is derived from the area under the spectral peak of the element.

**Fig 3:**
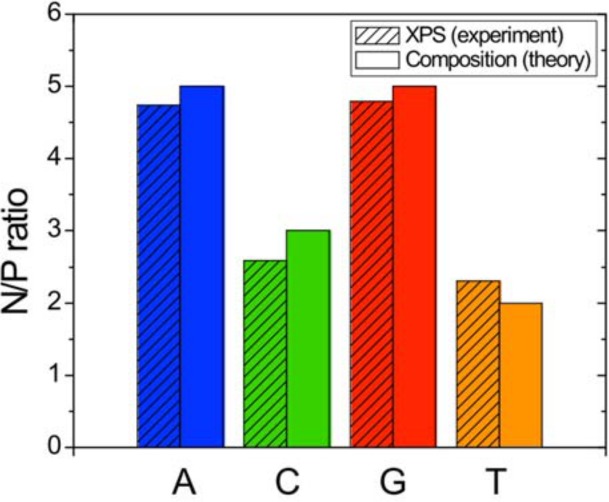
Nitrogen-to-phosphor ratio from XPS analysis of A, C, G and T 20mers.

**Fig 4:**
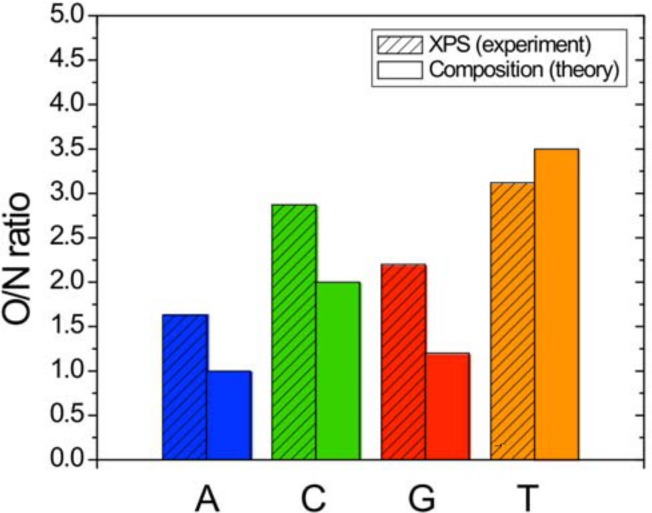
Oxygen-to-nitrogen ratio from XPS analysis of A, C, G and T 20mers.

Figs 3 and 4 show the quantitative analysis of the acquired XPS spectra for the four nucleotide samples. Fig 3 displays a bar graph of the N/P elemental ratios as derived from the experimental XPS spectra and compares them to the theoretical elemental nucleotide composition ratio. The measured N/P ratios agree well with theory and can be used to identify the pyrimidine samples C and T with 3 and 2 Nitrogen atoms per base, respectively.

The purines, A and G, both contain 5 nitrogen atoms and cannot be distinguished by the N/P ratio alone. Fig 4 shows the O/N ratio in comparison to the theoretical ratios. Here, we find an acceptable qualitiative agreement with the expected ratios for the XPS spectra albeit with an excess of oxygen, likely due to oxygen-containing contaminations [8,9]. The amount of oxygen is different between the purine bases (Adenine contains only the 4 oxygen atoms from the phosphate group while Guanine has an additional oxygen atom present in the nucleobase) so the O/N ratio is unique for each individual base.

**Fig 5:**
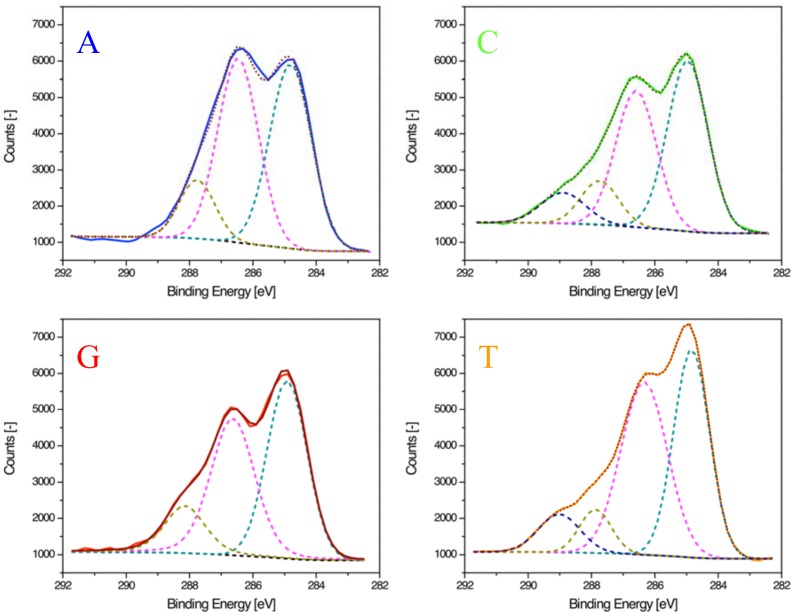
High-resolution carbon 1s XPS spectra for single-stranded homopolymeric 20mers.

Carbon 1s spectra with deconvoluted components for each individual base are shown in Fig 5. Each base contains multiple peaks from hydrocarbons (C-C and C-H) and carbon bound to oxygen (C-OH) that are present in either the nucleobase or the sugar backbone. In addition, each nucleobase contains additional carbon species like carbon bound to nitrogen (C-N, N-C-N and N = C(-N)-N), amide carbon (N-C = O) and urea carbon (N-C (= O)-N). The compositions of these carbon species are unique for each base but interpretation of individual components is complex. Nonetheless high binding energy species like urea carbons (present in C and T) are noticeable as a shoulder at 289 eV. In addition, the shape of the spectra for A and G differ from each other with well separated peak maxima (286.4 and 285.0 eV).

Previous reports [10–12] have analyzed carbon 1s spectra of DNA and have fitted the data with peaks at approximately 285, 286.5, 288 and 289 eV. We assigned these binding energies to the various carbon species and found a reasonable agreement with earlier studies and expected peak ratios.

### Auger Electron Spectroscopy (AES)

**Fig 6:**
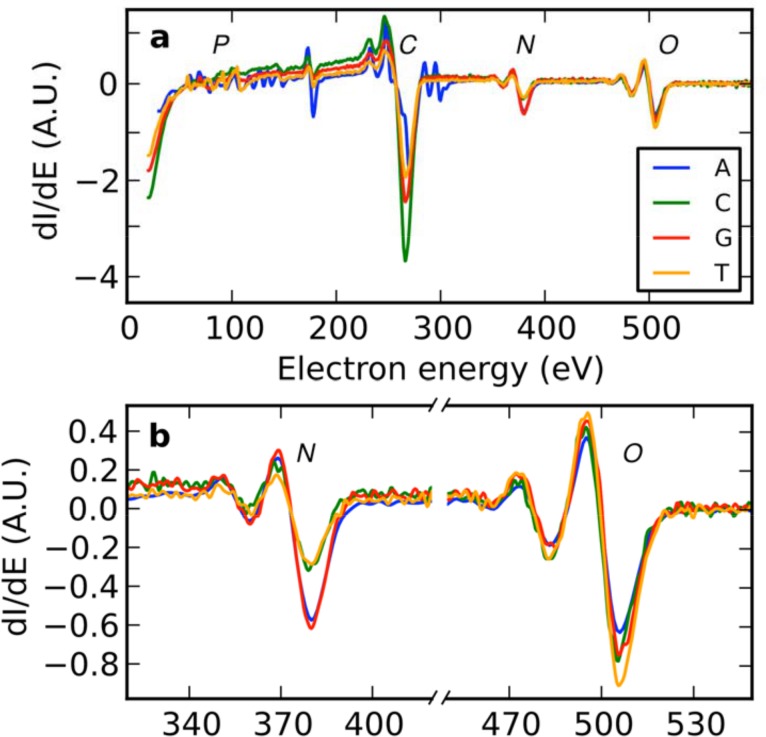
Auger spectra for single-stranded homopolymeric 5mers. Peak positions for phosphor (LMM 120 eV), carbon (KLL 272 eV), nitrogen (KLL 379 eV) and oxygen (KLL 503 eV) are marked with the respective elemental symbols. Spectra are normalized to obtain the expected ratio of oxygen (A:C:G:T = 5:6:6:7) as shown in Fig 1. Panel a shows an overview of detected electrons in the 30–600 eV range, and panel b shows zoom-ins of the nitrogen and oxygen peaks.

We used AES to analyze individual samples of homopolymeric 5mers that were prepared by electrostatic adsorption onto gold coated surfaces modified with aminoundecanethiol. We acquired numerically differentiated [13] AES spectra of A, C, G, and T covered specimens, and the prominent peaks for carbon, nitrogen, and oxygen are detected in the Auger spectra of all DNA specimens, as shown in Fig 6. The peak at 179 eV can be attributed to tantalum, the sample holder material. Peak heights were determined as the difference between the maximum and the minimum of the derivative spectrum around the expected peak position. While the phosphor peak has a very low signal, the intensity of the carbon peak varies significantly, possibly due to contamination which is further exacerbated by the primary electron beam. The carbon peak is also present and of comparable height on the reference specimen, whereas nitrogen and oxygen peaks measured on the reference specimen are approximately 80% to 90% smaller than the ones measured on DNA. We thus focus on the signal originating from nitrogen and oxygen (Fig 6B). Although the Auger peak height for one element is roughly proportional to its abundance within the sample, the peak heights of different elements are not necessarily comparable due to different cross-sections for Auger electron emission. Nevertheless, the ratio of the O/N AES peak heights can still serve as a measure to identify the nucleotide immobilized on the specimen.

**Fig 7:**
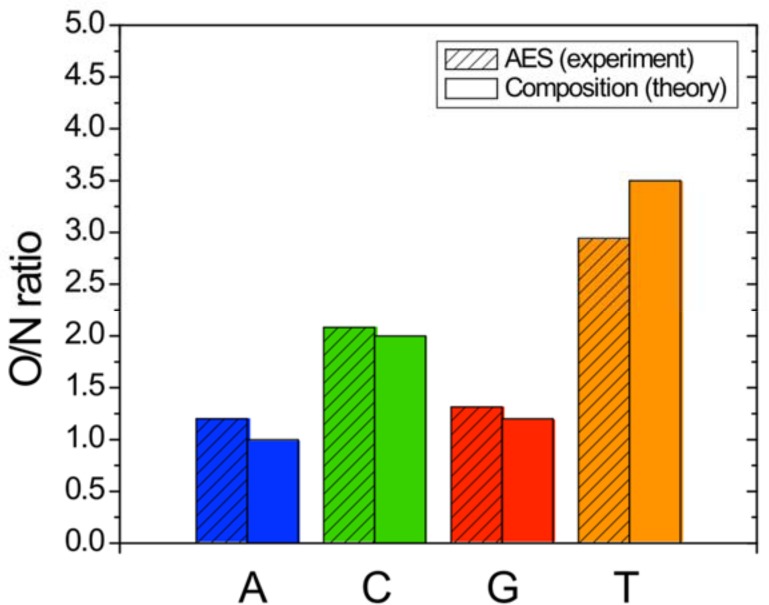
Oxygen-to-nitrogen ratio from Auger analysis of A, C, G and T 5mers.

Fig 7 shows the quantitative analysis of the acquired Auger spectra for the four nucleotide samples. It displays bar graphs of the O/N peak height as derived from the experimental Auger spectra and compares them to the theoretical elemental nucleotide composition ratio. Purines (A and G) and Pyrimidines (C and T) are clearly distinguishable, and C and T can be easily held apart as well. However, the contrast between A and G is more subtle and may be challenging for sequencing by imaging. As an additional pathway to discriminate between A and G, future studies will examine the peak shapes and energy shifts between A and G covered samples.

## Discussion

**Fig 8:**
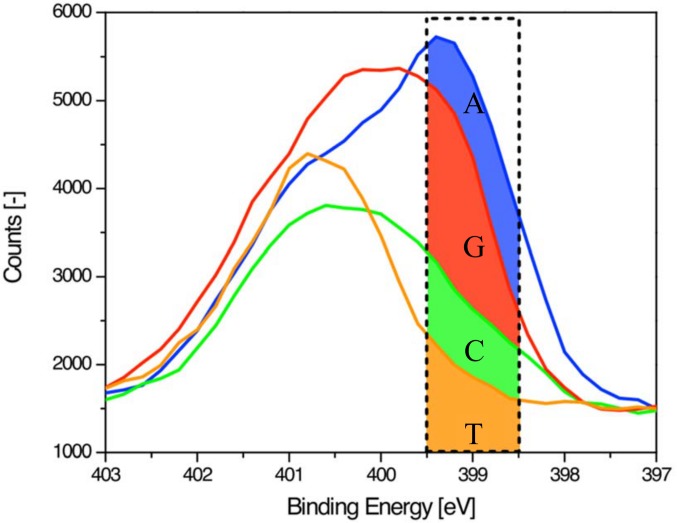
High-resolution nitrogen 1s XPS spectra for single-stranded homopolymeric 20mers. The dashed window centered at 399 eV highlights the difference in the amount of conjugated nitrogen between the various nucleotides.

Further detailed analysis of the XPS spectra reveals that the nitrogen spectrum itself may be sufficient to unambiguously distinguish the individual bases. This realization can be understood by focusing on the small energy window centered around 399 eV marked by a dashed box in the zoomed-in view of the individual nitrogen 1s spectra in Fig 8. Previous studies [10,11] have divided the nitrogen spectra for DNA bases into two binding energy regions, one above 400 eV and the other one around 399 eV. The higher binding energy region is attributed to single-bonded (non-conjugated sp^3^) amines and the lower region to double-bonded (conjugated sp^2^) imino species. T has 2 single-bonded amines and accordingly a narrow spectra containing only the high binding energy peak is present in the sample. All the other samples contain both high and low energy peaks as expected from their mixed composition of both single and double-bonded nitrogen. Accordingly, the nitrogen signals from the narrow energy window centered at 399 eV can be used to assign the individual bases. Within this energy window, nitrogen signals are increasing in nearly equal steps, starting with the minimum nitrogen content for T, followed by increased content for C and G, and reaching a maximum for A. The roughly linear dependence on the nitrogen signal is attributed to the aforementioned double-bonded nitrogen present in differing amount in the individual bases: T has no double-bonded nitrogen, C has one, G has two and A has three (see Fig 1). The integrated counts for the four bases, T, C, G, and A are approximately 1900, 2800, 4200, and 5100, respectively. Subtraction of 1900 (for T) results in remaining counts of 0, 900, 2300 and 3200 each for T, C, G and A. This corresponds to the attained amount of double-bonded nitrogen for each base. We also performed peak-fitting on the individual nitrogen spectra (S2 Fig) and found that the obtained peak ratios qualitatively follow the predicted ratios with a minor overrepresentation for the lower binding energy peak at 399 eV, comparable to a previous report [10]. The nominal pair contrast, defined here as a ratio of the difference and the sum of the individual base signals, obtained from the XPS measurements on bulk DNA, is estimated from the integral of the counts in Fig 8 over a 1 eV window about the energy of 399 eV. Using the integrated counts listed above, the nominal pair contrast becomes 0.2 and 0.5 for the GC and AT base pairs, respectively. For single strand DNA specimens, the signal from the substrate can potentially weaken the contrast. However, we believe that the potential reduction of the contrast can be in part mitigated by a proper choice and cleanliness of the substrate, in particular by avoiding potential Nitrogen contamination and compounds with elemental signals that could overlap the Nitrogen peaks.

We note that the correlation between the increasing signal levels at 399 eV and the amount of double-bonded nitrogen is also reflected in the binding energy at peak maximum of the entire nitrogen spectra. The peak maximum position is related to the amount of individual nitrogen species within each base. A lower peak maximum is found in purine bases which have a higher content of double-bonded nitrogen. The purine A with the highest amount of double-bonded nitrogen has the lowest peak maximum (399.4 eV) followed by G (399.8 eV), C (400.6 eV) and T (400.8 eV). This suggests that in addition to the spectral count at 399 eV, the peak maxima and the shape of the spectra (characteristic shoulder for A and narrow peak for T) can be used to distinguish the individual bases. Binding energies are known to increase with film thickness due to extra-atomic relaxation and charging effects [14, 15]. Thus for a thinner film consisting of individually aligned DNA strands a uniform shift to lower peak binding energies is expected without significant changes between the four nucleobases. These films are also expected to be thin (< = 2 nm) with minimal effects caused by the orientation of the DNA strands [10]. Nitrogen 1s photoelectrons have kinetic energies on the order of 1000 eV, and simulations show that energy windows as large as 1 eV can be used for imaging while maintaining sub-nanometer resolution [7]. Consequently, we believe that in the XPS imaging mode we can use either a single energy-filtered image of the nitrogen peak at the energy level of 399 eV or a combination of the shape and peak maxima of the nitrogen 1s spectra to distinguish individual bases.

In the XPEEM imaging mode, electrons from a small energy window are selected with which to form the image in order to deliver analytical information about the specimen such as its elemental composition. The application of the optimum aperture to the wide angular distribution of the photo-emitted electrons combined with the additional filtering by the energy window can significantly reduce the fraction of the electrons that reach the detection plane when compared to the collection efficiency of the reflected electrons in the LEEM imaging mode. However the resulting loss in the intensity is partially recovered by the greater nominal contrast of the X-ray signal and by the introduction of the pentode MAC, which significantly increases the optimum aperture angle as well as the tolerable energy spread. In order to achieve realistic acquisition times, the intensity of the illuminating X-ray beam needs to be increased by focusing the illuminating X-ray beam to provide a close match to our desired field of view (a few μm) and by utilizing a high brilliance X-ray source, e.g. available in a synchrotron beam line. Radiation damage could potentially be an issue but a related XPS [11] study found that the Nitrogen region of interest did not show any X-ray damage for a continuous X-ray exposure for 4 hours and we expect our exposure time to be significantly shorter. A careful optimization balancing the acquisition time and X-ray beam intensity will be required.

## Conclusions

We have presented XPS and AES investigations of DNA homopolymers that demonstrate two feasible contrast mechanisms that can distinguish individual nucleotides without labels. We have shown experimentally that the nucleotide element ratios and bonding states can be used to distinguish the individual bases. Detailed, quantitative analysis of the XPS and AES spectra shows that the elemental composition and bonding states are in good qualitative agreement with theory and yields an appreciable nominal contrast of 0.2 and 0.5 for the GC and AT base pairs. The experimental contrast obtained in XPS and AES holds promise for sequencing by imaging in an electron microscope. This can be achieved by integrating the XPS and AES imaging modes into an aberration-corrected low energy electron microscope to enhance their lateral resolution to the sub-nanometer range and explore their elemental and chemical specificity to distinguish the individual nucleotides. This approach thus has promise to significantly improve the performance of a DNA sequencing tool based on direct imaging and will be suitable for a wide range of applications in the biosciences, material sciences, and nanotechnology, where nanometer scale resolution and analytical capabilities are required.

## Materials and Methods

### Specimen preparation

Gold surfaces were obtained by sputtering the gold onto silicon wafers using chromium as an intermediate adhesive layer (Stanford Nanofabrication Facility). Prior to use, gold substrates were diced into 50 mm^2^ pieces (~7x7 mm^2^) and cleaned in UV-Ozone (Jelight UVO-cleaner 42) for 15 minutes. This was followed by immediate immersion in a 1 mM aminoundecanethiol (Sigma Aldrich) solution in ethanol for overnight incubation. The substrates were subsequently washed extensively with ethanol and dried in a stream of argon. Homopolymeric oligonuclotides (5mers and 20mers purchased from Integrated DNA Technologies) were dissolved in deionized water. All samples were prepared by electrostatic adsorption of oligonuclotides onto self-assembled monolayers (SAMs) of aminoundecanethiol. XPS samples containing homopolymeric 20mers were prepared by spotting 10 μL droplets of a 100 μM oligo solution directly onto the SAM -layer and left to dry overnight in a sealed NaCl saturated Teflon chamber. The thickness of the DNA films were adjusted by the number of droplets spotted onto the samples. Typically 2–4 droplets were used for each individual sample. Samples used for Auger measurements were prepared in a similar manner with the exception that the oligomer solution contained 5mers diluted to 10 μM. The use of lower concentration and shorter oligomers resulted in thinner DNA films (approximately 10 monolayers instead of a few 100 monolayers) and was found to be favorable to mitigating charging effects. Surface charging, specimen thickness and substrate signal affect to a different degree the experimental results in XPS, Auger and LEEM, with XPS being the least and LEEM the most sensitive to charging. While MAD-LEEM would not only offer spatial resolution but also compensate charging for all these imaging modes, our currently available instrumentation required slightly different DNA film conditions to balance the charging and the presence of parasitic signal from the substrate and surface contaminations.

### XPS

XPS is a quantitative spectroscopic technique that measures the elemental composition and the chemical and electronic states of a specimen. The spectra are obtained by irradiating the material with a beam of X-rays, while simultaneously measuring the kinetic energy and number of electrons that escape the specimen. In this method, low energy electrons are measured, giving rise to shallow depth and high chemical sensitivity values, which are respectively on the order of nanometers and of about 0.1% atomic concentration. XPS measurements were performed on a commercial XPS System (PHI Versaprobe) equipped with a monochromatic Al Kα source and a hemispherical electron energy analyzer using a spot size of 100 μm. The acquisition time for the individual Nitrogen spectra was 100 s. The maximum X-ray photon flux is estimated to be approximately 10^11^ photons/(mm^2^ s), resulting in an average X-ray photon dose of ~ 10 photons / nm^2^ for the exposure time of 100 s. Survey scans were performed on all samples using a pass energy of 100 eV. Survey scans consistently identified the expected elements (C, O, N, P) and occasionally trace amounts of Au (from the underlying substrates) or adventitious elements (typically Na that is believed to be present as a counter ion to the phosphate in the DNA). Samples with a Au signal exceeding 1% in the survey scans were re-spotted with additional DNA to make a thicker DNA film. Electrons detected from the Au layer have higher kinetic energy (and a larger escape depth) than any electrons detected from the aminoundecanethiol layer (C or N). Thus the absence of Au peaks in the survey spectra is a confirmation that neither nitrogen nor carbon from the aminoundecanethiol layer are included in the analysis. High resolution XPS spectra were obtained using a pass energy of 20 eV. All samples identified the common phosphor 2p peak within a narrow window of 0.2 eV. Consequently we report all XPS peaks referenced to this phospor peak (aligned at 133.6 eV in accordance with previous studies) [14]. High resolution XPS peaks were fit using Multipak software. A convolution of Lorentzian and Gaussian line shapes was used to fit the individual peaks while the background was modeled using a combination of Shirley and linear functions. Individual peak positions were kept fixed within +/- 0.2 eV for all samples, and the full width at half maximum (fwhm) converged to consistent values between 1.2 eV (for C 1s) and 1.8 eV (for N 1s) throughout the series without restrictions and are consistent with common ranges for polymer-like materials.

### AES

In Auger Electron Spectroscopy the sample is excited with a primary electron beam with a fixed energy of several keV and an energy spectrum of secondary electrons is recorded. The primary beam excites secondary electrons by several mechanisms, including Auger electron emission. In this process, a tightly bound electron from a core level (e. g. the K-shell) is emitted. Another electron from a higher level (e. g. L1) can gain energy by filling the resulting hole. This energy gain can either be released as a photon or it can be transferred to a third electron bound to the atom (e. g. in the L2,3-shell). If the transferred energy exceeds the binding energy, then an Auger electron can be emitted (a KLL emission). As the transferred energy and thus the kinetic energy of the emitted Auger electron is dependent on the electron binding energy differences, the Auger electron energy spectrum is element specific and also sensitive to the chemical environment to some degree. Auger electron energy spectra are usually differentiated for analysis to remove the large background of secondary electrons [13]. AES measurements were performed on a commercial AES system (Perkin-Elmer PHI 10–155) operating at 3 keV and with an emission current of 1mA. The beam is focused at the sample into a spot of approximately 1 mm^2^ with a maximum beam current of 50 μA. Its cylindrical mirror analyzer has an energy resolution of 0.5 eV or less for the applied energy range. Spectra are composed of five averaged passes with a step size of 500 meV and a dwell time of 100 ms at each energy step, with a total acquisition time of 960 s per spectrum. The current density at the specimen is 5 x 10^−17^ A / nm^2^, and we estimate the average electron dose to be approximately 300,000 electrons / nm^2^. All differential spectra have been normalized to obtain the expected ratio of oxygen (A:C:G:T = 5:6:6:7) and the linear background based on the featureless stretch between 600–900 eV in the AES spectra has been subtracted.

### DNA preparation for sequencing by electron microscopy imaging

The challenge for imaging a sequence of DNA does not remain solely with the necessary resolving power of the electron microscope. First, DNA has to be removed from its natural environment, an aqueous solution, and subsequently deposited onto a substrate in a manner suitable for imaging: a process referred to as linearization. Here we outline the requirements for the preparation of DNA for sequencing by imaging and give an overview of the state of the art. This outline is the basis of the model for the presented contrast simulations.

If DNA could be extruded and linearized onto a substrate, there would still remain the question of what form DNA takes outside of an aqueous environment. In its natural form, known as B-DNA, the polymer comprises two helical, intertwined strands. The two strands contain a complementary sequence of nucleotides with the bases on each strand held together by a sugar-phosphate backbone and with each base forming hydrogen bonds with its complement on the other strand. If the polymer were to retain its natural state when extruded from solution, then the tight spacing between the base pairs characterized by a rise per base pair of γ = 0.34 nm would present the primary challenge for resolving them. In addition, there is the issue of differentiating the base-to-base intensity contrast, which provides the sequence information, from the competing signal that would arise from the helical, intertwined geometry, which repeats itself every h = 3.4 nm or 10 base pairs.

A number of groups have worked on related problems and there exists a body of literature which helps to assess these questions. In the 1990’s, experiments [16,17] were performed in which DNA in solution was grafted onto a silinated substrate (surface treated with silicon-hydrides). Upon extrusion of the substrate from the solution, the anchored DNA was found to remain attached to the surface in a manner that did not form clumps. Furthermore, the DNA polymer was found to have stretched by about 50%. This phenomenon, dubbed molecular combing, was attributed to the force associated with the surface tension of the air-water meniscus pulling on the DNA molecule. Estimates of this force were found to be two orders of magnitude greater than the entropic forces keeping the DNA polymer in a random coil configuration, but smaller than the force required to break a covalent bond. Furthermore, it was noted that because the force was localized at the air-water interface, it acted identically on each base pair leaving the solution, thereby stretching the polymer uniformly.

The molecular combing experiments were complemented by experiments on DNA micro-manipulation using optical tweezers and magnetic beads to twist and stretch DNA [18–20]. These studies have shown that a small force of order 1 pN is able to counter the effects of entropy to nearly stretch DNA to its contour length. Beyond this point, the force rises abruptly to stretch the bonds holding DNA in its normal B-form. When the force reaches 60-to-70 pN, DNA (that is free to twist) undergoes a phase transition where it stretches at near constant force to approximately 1.7 times its contour length. This new form, labeled as S-DNA, is believed to describe the stretched DNA in the molecular combing experiments.

While there have emerged successful, discrete and continuous chain models [21,22] to describe the force-extension curve of DNA from its entropic and elastic regions all the way through its phase transition (with an Ising model), there remains some ambiguity regarding the actual form of overstretched S-DNA. S-DNA cannot be a complete unwinding of the DNA helix. A geometric calculation of the contour length of the sugar-phosphate backbone helix shows that an unwound DNA ladder would extend by a factor of √ (1+ (2πa ∕ h)2) ≅ 2.1, not 1.7, where a = 1 nm is the helix radius.

Experimental findings [23] of suppression of the phase transition when DNA is twist-constrained have motivated a helical model of S-DNA that has a reduced winding with a helical turn of h = 22 nm holding approximately 37.5 base pairs to give a rise per base pair of γ = 0.59 nm (providing the 70% increase from the value in B-form). However, demonstrations of a reduction in the force required for the phase transition in chemical conditions that destabilize the DNA double helix have led to compelling thermodynamic arguments suggesting that S-DNA is composed of long stretches (of order 100 base pairs) of “melted”, single-stranded DNA held together by local regions of remnant base pairing [24].

## Supporting Information

S1 Fig High-resolution P 2p XPS spectra for single-stranded homopolymeric 20mers. All spectra have been peakshifted to 133.6 eV and scaled to the same height. The obtained peakshift and scaling factor were used for normalization of all other elements throughout each sample.
(DOCX)

S2 Fig Peak-fitted high-resolution N 1s XPS spectra for single-stranded homopolymeric 20mers. The data for all samples are fit with one peak for single-bonded nitrogen at 400.6 eV. In addition the Adenine, Cytosine and Guanine samples are fit with a second peak at 399.5 eV corresponding to double-bonded nitrogen.
(DOCX)
