# Supplementary material for: Nucleotide-Specific Contrast for DNA Sequencing by Electron Spectroscopy
Source: PLoS One. 2016 May 5;11(5):e0154707. doi: 10.1371/journal.pone.0154707 (PMC4858156; doi:10.1371/journal.pone.0154707)
Supplement: S1 Fig — All spectra have been peakshifted to 133.6 eV and scaled to the same height. The obtained peakshift and scaling factor were used for normalization of all other elements throughout each sample. (DOCX) [file pone.0154707.s001.docx]

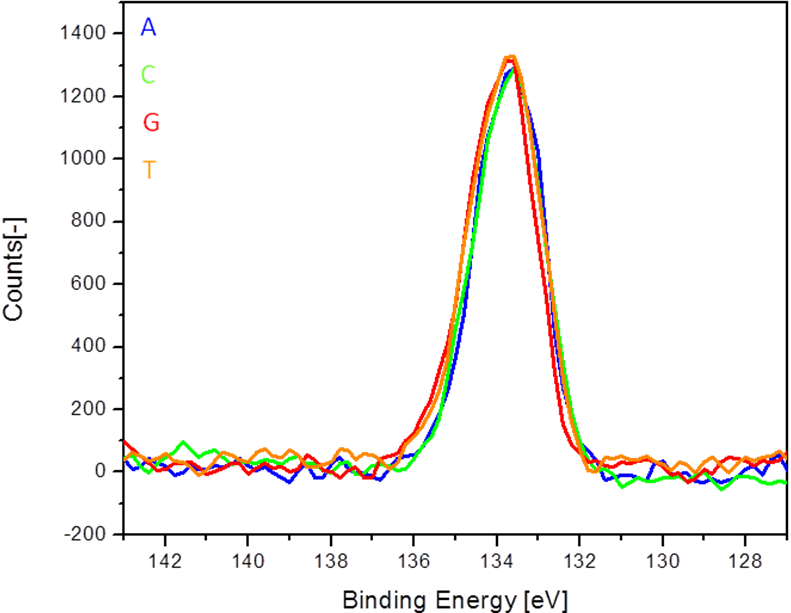


**S1 Fig.** High-resolution P 2p XPS spectra for single-stranded homopolymeric 20mers. All spectra have been peakshifted to 133.6 eV and scaled to the same height. The obtained peakshift and scaling factor were used for normalization of all other elements throughout each sample.
