# Supplementary material for: Nucleotide-Specific Contrast for DNA Sequencing by Electron Spectroscopy
Source: PLoS One. 2016 May 5;11(5):e0154707. doi: 10.1371/journal.pone.0154707 (PMC4858156; doi:10.1371/journal.pone.0154707)
Supplement: S2 Fig — The data for all samples are fit with one peak for single-bonded nitrogen at 400.6 eV. In addition the Adenine, Cytosine and Guanine samples are fit with a second peak at 399.5 eV corresponding to double-bonded nitrogen. (DOCX) [file pone.0154707.s002.docx]

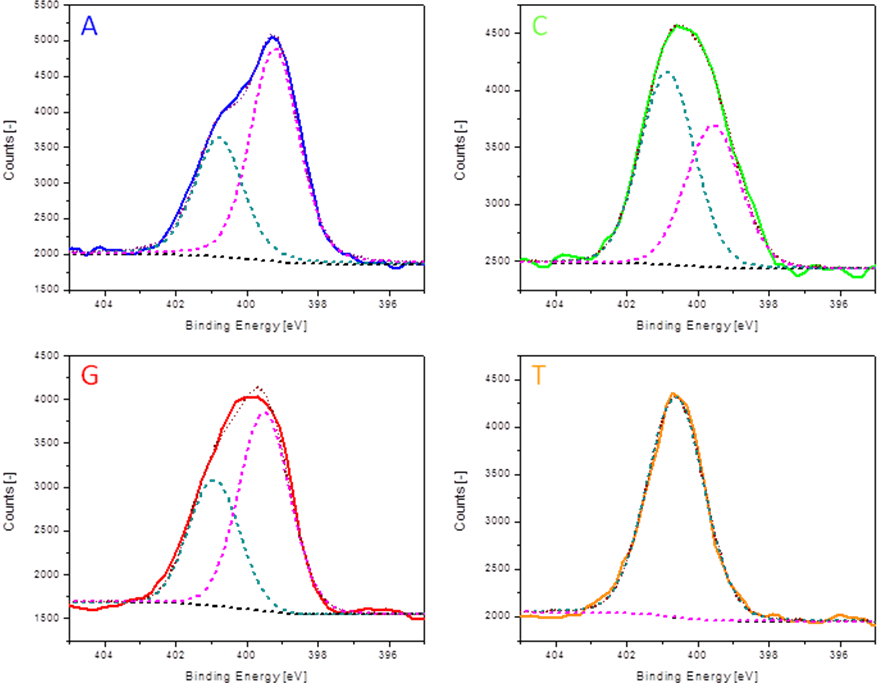


**S2 Fig.**  Peak-fitted high-resolution N 1s XPS spectra for single-stranded homopolymeric 20mers. The data for all samples are fit with one peak for single-bonded nitrogen at 400.6 eV. In addition the Adenine, Cytosine and Guanine samples are fit with a second peak at 399.5 eV corresponding to double-bonded nitrogen.
